# Evaluating the ototoxicity of an anti-MRSA peptide KR-12-a2^☆^

**DOI:** 10.1016/j.bjorl.2017.05.002

**Published:** 2017-05-31

**Authors:** Chung Man Sung, Hong Chan Kim, Yong Beom Cho, Song Yub Shin, Chul Ho Jang

**Affiliations:** a Chonnam National University Medical School, Department of Otolaryngology, Gwangju, South Korea; b Chosun University, School of Medicine, Graduate School and Department of Cellular & Molecular Medicine, Gwangju, South Korea

**Keywords:** KR-12-a2, anti-MRSA peptide, Topical application, Ototoxicity, KR-12-a2, Peptídeo Anti-MRSA, Aplicação tópica, Ototoxicidade

## Abstract

**Introduction:**

Methicillin-resistant *staphylococcus aureus* is an emerging problem for the treatment of chronic suppurative otitis media, and also for pediatric tympanostomy tube otorrhea. To date, there are no effective topical antibiotic drugs to treat methicillin-resistant *staphylococcus aureus* otorrhea.

**Objective:**

In this study, we evaluated the ototoxicity of topical KR-12-a2 solution on the cochlea when it is applied topically in the middle ear of guinea pigs.

**Methods:**

The antimicrobial activity of KR-12-a2 against methicillin-resistant *staphylococcus aureus* strains was examined by using the inhibition zone test. Topical application of KR-12-a2 solution, gentamicin and phosphate buffered saline were applied in the middle ear of the guinea pigs after inserting ventilation tubes. Ototoxicity was assessed by auditory brainstem evoked response and scanning electron microscope examination.

**Results:**

KR-12-a2 produced an inhibition zone against methicillin-resistant *staphylococcus aureus* from 6.25 μg. Hearing threshold in the KR-12-a2 and PBS groups were similar to that before ventilation tube insertion. However, the gentamicin group showed elevation of the hearing threshold and there were statistically significant differences compared to the phosphate buffered saline or the KR-12-a2 group. In the scanning electron microscope findings, the KR-12-a2 group showed intact outer hair cells. However, the gentamicin group showed total loss of outer hair cells. In our experiment, topically applied KR-12-a2 solution did not cause hearing loss or cochlear damage in guinea pigs.

**Conclusion:**

In our experiment, topically applied KR-12-a2 solution did not cause hearing loss or cochlear damage in guinea pigs. The KR-12-a2 solution can be used as ototopical drops for treating methicillin-resistant *staphylococcus aureus* otorrhea; however, further evaluations, such as the definition of optimal concentration and combination, are necessary.

## Resumo

**Introdução:**

O *Staphylococcus aureus* resistente à meticilina é um problema emergente não só para a otite média supurativa crônica, mas também para casos de otorreia crônica em crianças com tubo de ventilação. Até o momento, não há antibióticos tópicos efetivos para a otorreia causada por *staphylococcus aureus* resistente à meticilina.

**Objetivo:**

Nesse estudo, avaliamos a ototoxicidade da solução tópica de KR-12-a2 na cóclea quando aplicada topicamente na orelha média de cobaias.

**Método:**

A atividade antimicrobiana de KR-12-a2 contra cepas de *staphylococcus aureus* resistente à meticilina foi avaliada utilizando-se o teste de zona de inibição de crescimento. Foram aplicados na orelhas médias de 3 grupos de cobaias, ou solução tópica de KR-12-a2, ou gentamicina ou solução salina tamponada com fosfato após timpanostomia. A ototoxicidade foi avaliada através do exame auditivo de potencial evocado auditivo de tronco encefálico e por microscopia eletrônica de varredura.

**Resultados:**

O KR-12-a2 produziu uma zona de inibição contra o *staphylococcus aureus* resistente à meticilina a partir de 6,25 μg. Alterações do limiar de audição no grupo KR-12-a2 e no grupo com solução salina foram semelhantes aos observados antes da inserção do tubo de ventilação. No entanto, o grupo gentamicina apresentou um limiar auditivo mais elevado, estatisticamente significativo em comparação ao grupo solução salina ou ao grupo KR-12-a2. Nos achados da microscopia eletrônica, o grupo KR-12-a2 apresentou células ciliadas externas intactas. No entanto, o grupo gentamicina apresentou perda total das células ciliadas externas. Em nosso experimento, a solução de KR-12-a2 aplicada topicamente não causou perda auditiva ou dano coclear em cobaias.

**Conclusão:**

Em nosso experimento, a solução de KR-12-a2 aplicada topicamente não causou perda auditiva ou dano coclear em cobaias. A solução de KR-12-a2 pode ser utilizada como gotas otológicas para o tratamento da otorreia causada por *staphylococcus aureus* resistente à meticilina; no entanto, são necessárias outras avaliações, para a definição da concentração e das associações ideais.

## Introduction

Antibiotic resistance is rapidly becoming a clinically significant problem to the healthcare system. Treatment of chronic otitis media caused by bacteria, resistant to multiple antimicrobial agents, has emerged as one of the greatest challenges faced by clinicians. Consequently, Methicillin-Resistant *Staphylococcus Aureus* (MRSA) infections cannot be treated with commercially available ciprofloxacin ototopical drops.^1^, ^2^ The prevalence of MRSA in chronic suppurative otitis media (CSOM) is known to be more than ∼60–70% among *S. Aureus* isolates from hospitals in Korea.^3^ Besides CSOM, MRSA is an emerging problem related to chronic pediatric tympanostomy tube otorrhea. To date, standard treatment protocols have not been developed, and there is a diverse spectrum of reported medical treatments. The additional use of oral antibiotics such as linezolid,^4^ trimethoprim-sulfamethoxazole,^5^ fusidic acid,^6^ or rifampin to treat MRSA otorrhea may be helpful, but after systemic administration, the effective bioavailable concentration in the middle ear to eradicate infection cannot be accurately measured or described. Ototopical drug delivery presents a highly promising alternative to oral antibiotics for the administration of therapeutics to the middle ear.

The use of vancomycin for the treatment of MRSA infections has increased.^7^ One of the authors of this paper used topical vancomycin to treat patients with MRSA CSOM and obtained good results.^8^ However, the concern regarding the use of vancomycin is the possible emergence of vancomycin-resistant strains such as Vancomycin-Intermediate *S. aureus* (VISA) and Vancomycin-Resistant *Enterococcus* (VRE). In clinical settings, its less frequent use may facilitate decreases in the appearance of vancomycin-resistant strains. Therefore, it is necessary to develop new antimicrobial compounds against the MRSA. Recently, Antimicrobial Peptides (AMPs) have been considered as novel antibiotics against drug-resistant microorganisms, such as MRSA, and some of them are being evaluated in clinical trials.^9^ AMPs are fundamental components of the mammalian innate immunity that control microbial infections and represent the first line of defense against different pathogens on epidermal and mucosal epithelial surfaces. AMPs play a significant role in host innate immunity defense to prevent first infections^10^ and are produced by macrophages, blood cells, and epithelial cells in mammalians. The AMPs identified in humans include members of the cathelicidin, defensin, thrombocidin, and histatin families.^11^

Expression of an AMP in the skin of the external auditory canal skin and in the middle ear has been reported.^12^, ^13^, ^14^ Human cathelicidin LL-37 is a 37 residue-long cationic, amphipathic α-helical antimicrobial peptide.^15^ Besides its direct bactericidal action, LL-37 is known to have a potent Lipopolysaccharide (LPS) neutralizing activity in various cell types.^16^, ^17^, ^18^ KR-12 (residues 18–29 of LL-37) is the smallest peptide of human cathelicidin LL-37 possessing antimicrobial activity.^19^ Recently, we developed novel KR-12 analogs, designed based on the structure–activity analysis of KR-12 (KRIVQRIKDFLR-NH_2_),^20^ which showed potent antimicrobial and anti-endotoxic activities.

Purulent otorrhea is a common disease in patients suffering from CSOM. Patients with CSOM and MRSA infection generally have a chronic inflammation of the middle ear and mastoid, with a persistent perforation of the tympanic membrane, associated with recurrent otorrhea. Ototopical medication leads more likely to the use of lower doses compared to systemic antibiotics. Moreover, the ability of systemic antibiotics for the treatment of infections associated with infected implants in the middle ear is not well elucidated. However, ototopical drops against MRSA are not commercially yet available. In this study, we evaluated the ototoxic effect of a topical solution of KR-12-a2 (KRIVQRIKKWLR-NH_2_), a KR-12 analog, on the cochlea after topical application in the middle ear of guinea pigs.

## Methods

### Peptide synthesis

**Figure 1:**
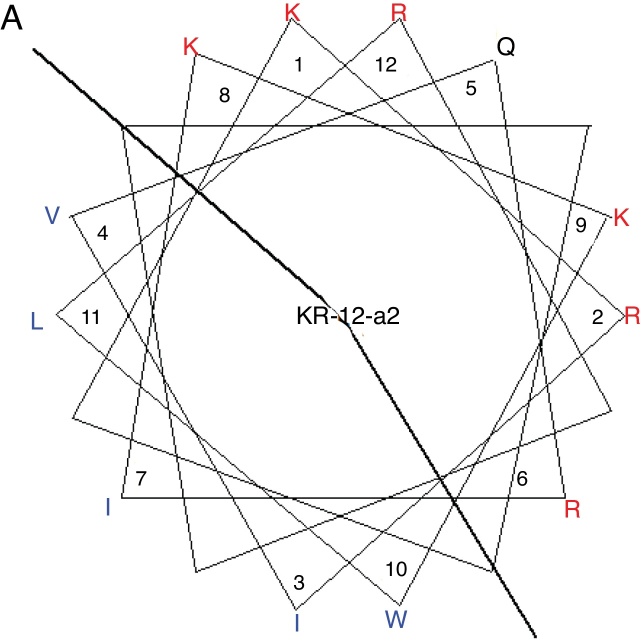

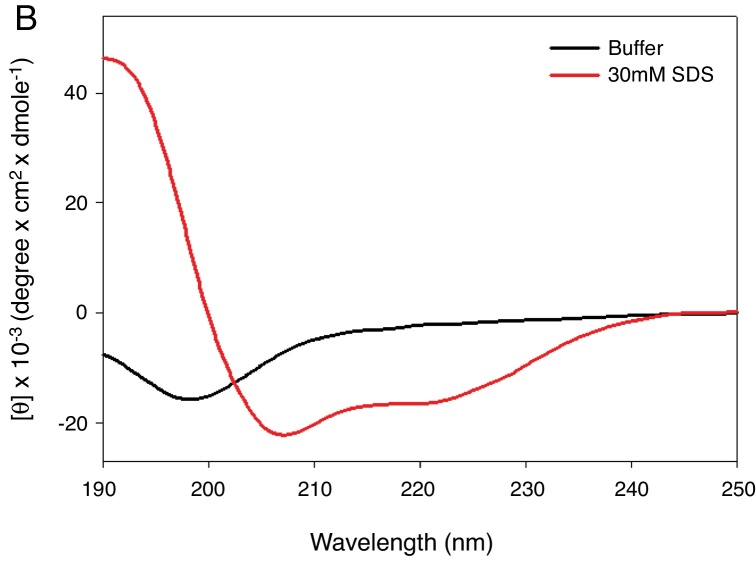
(A) The α-helical wheel diagrams for KR-12-a2. The positively charged and hydrophobic amino acids are represented using red and blue color, respectively. The line indicates the interface between the hydrophobic and the positively charged face. (B) Circular Dichroism (CD) spectra of the peptides in 10 mM sodium phosphate buffer, pH 7.2, and 30 mM Sodium Dodecyl Sulfate (SDS) micelles.

KR-12-a2 was prepared as described previously by us.^19^ Briefly, the preparation of the KR-12-a2 involves the standard 9-Fluorenylmethoxycarbonyl (Fmoc) based solid-phase synthesis technique on rink amide 4 methylbenzhydrylamine resin (0.54 mmoL/g). Dicyclohexylcarbodiimide (DCC) and N-hydroxybenzotriazole (HOBt) were used as coupling reagents, and a 10-fold excess of Fmoc-amino acids was added during every coupling cycle. After cleavage and deprotection with a mixture of trifluoroacetic acid/water/thioanisole/phenol/ethanedithiol/triisopropylsilane (3.26 mL/0.2 mL/0.2 mL/0.2 mL/0.1 mL/0.04 mL) for 2 h at room temperature, the crude peptide was repeatedly extracted with diethyl ether and purified via Reverse-Phase High-Performance Liquid Chromatography (RP-HPLC) on a preparative Vydac C_18_ column (20 mm × 250 mm, 300 Å, 15 mm particle size) using an appropriate 0–90% water/acetonitrile gradient in the presence of 0.05% trifluoroacetic acid. The final purity of the peptides (>95%) was assessed by RP-HPLC on an analytical Vydac C_18_ column (4.6 mm ×  250 mm, 300 Å, 5 mm particle size). The success of the synthesis of the peptide was confirmed via analysis using a matrix-assisted laser desorption/ionization, time-of-flight (MALDI-TOF) mass spectrometry (Shimadzu, Japan). As shown in its α-helical wheel projection (Fig. 1A), KR-12-a2 was designed to be amphipathic when folded into a *α*-helical structure, by converging the hydrophobic residues (Val, Leu, Ile, and Trp) to one side and the hydrophilic residues (Lys and Arg) to the other side of the helical axis. In addition, KR-12-a2 showed a characteristic α-helical CD spectrum with two dichroic minimal values at 208 and 222 nm and a positive band near 192 nm in 30 mM sodium Dodecyl Sulfate (SDS) micelles (membrane-mimicking environments) (Fig. 1B).

### Inhibition zone assay

MRSA was grown overnight for 18 h at 37 °C in 10 mL of LB broth and then 10 μL of this culture was inoculated into 10 mL of fresh LB and incubated for an additional 3 h at 37 °C to obtain mid-logarithmic phase organisms. The bacterial suspension (2 × 10^5^ CFU/mL in LB) was mixed with 0.7% agarose and poured into a 10 cm Petri dish. Ten-microliter aliquots of a serial dilution of KR-12-a2 were placed in each circle paper (≈6 mm in diameter) put on the agarose plates and then incubated at 37 °C overnight. The diameters of the bacterial clearance zones surrounding the circle paper were measured for the quantitation of inhibitory activities.

### Myringotomy and ventilation tube insertion and topical application

The experiments were performed in 15 young male guinea pigs (weighing 250–300 g each) with normal tympanic membranes. All animal experiments followed a protocol approved by the Committee for Animal Experimentation (CNU IACUC-H-2014-36). The guinea pigs were anesthetized using an intraperitoneal injection of zoletil and xylazine hydrochloride. The ear canals and the tympanic membranes of the guinea pigs were examined under an operating microscope. The possibility of ear infection was excluded by examining the external auditory canal and tympanic membrane of each guinea pig. After preoperative baseline hearing assessment, myringotomy and Mini Shah ventilation tube (inner diameter 0.76, JEDMED Co, St Louis, USA) insertion at the anterior portion of the tympanic membrane were performed. Topical application of 50 μL of KR-12-a2 solution (g/mL PBS diluted), (Group I, *n* = 5) using a 30 gauge, long needle attached syringe was performed into the middle ear via the ventilation tube. Phosphate-buffered saline (PBS) 50 μL (Group II, *n* = 5) and gentamicin 50 μL (40 mg/mL) (Group III, *n* = 5) were topically applied. Intra-tympanic administrations were performed twice daily for 7 days and the paper patch was applied after removal of tympanostomy tube.

### Auditory brainstem response (ABR) measurement before and after topical application

The guinea pigs were anesthetized using an intraperitoneal injection of zoletil and xylazine hydrochloride. The ABRs were recorded using an evoked potential system (Tucker-Davis Technologies, Florida, USA) and a Samsung computer. The stimuli were digitally synthesized using Siggen© software, and were presented through an insert earphone (ER-2, Etymotic Research, Inc. Illinois, USA). The acoustic stimuli, comprising a click and 4, 8, and 16 kHz tone bursts, were then produced. The intensity of the acoustic stimuli was expressed in Decibels (dB). The animals were presented with a stimulus intensity series, which was initiated at 90 Db SPL and it reached a minimum of 10 dB SPL. The stimulus intensity was progressively lowered in 10 dB decrements. Each average consisted of 500 stimuli presentations, with a 10 ms analysis time. The electrical activity was recorded via a platinum needle electrode inserted into the scalp at the vertex, and it was referenced to another needle electrode in a deep neck muscle. A third needle electrode in the pinna served as a ground. The intensities that appeared to be near threshold were repeated. The threshold was defined as the lowest intensity capable of producing a visually detectable, reproducible ABR response; the values were extrapolated to the nearest 5 dB assuming a log-linear fall off with decreasing intensity. At the threshold, the baseline-to-peak amplitude of the largest ABR component was reduced to <10% of that evoked by the 90 dB stimuli. The ABRs were assessed preoperatively and on the eighth day after topical application of the drug. Repeated measures analysis of variance (ANOVA) was used to evaluate the differences among the groups. A *p*-value <0.05 was considered statistically significant.

### Scanning electron microscopic observation of the cochlea

After the final ABR recordings, the anesthetized animals were perfused intra-cardially with 4% paraformaldehyde while under general anesthesia. The temporal bones were isolated and the peri-lymphatic spaces of the cochlea were gently perfused with 2.5% glutaraldehyde in 0.1 M PBS by performing cochleostomies at the round and oval windows. The bony capsules were removed to expose the organ of Corti, after which the specimens were post-fixed in 2.5% glutaraldehyde overnight at 4 °C. The specimens were washed three times in PBS and then post-fixed in 1% osmium tetroxide for 1 h at 4 °C. The organ of Corti specimens were then dehydrated through a graded series of ethanol solutions and were critical-point-dried using liquid carbon dioxide. These specimens of the organ of Corti were attached to aluminum SEM stubs with aluminum paint and then sputter-coated with gold-palladium. The Outer Hair Cells (OHC) of the organ of Corti were examined using a scanning electron microscope. The quantification of survived hair cells of all turns was performed.

## Results

### In vitro test of antimicrobial activity and cytotoxicity

**Figure 2:**
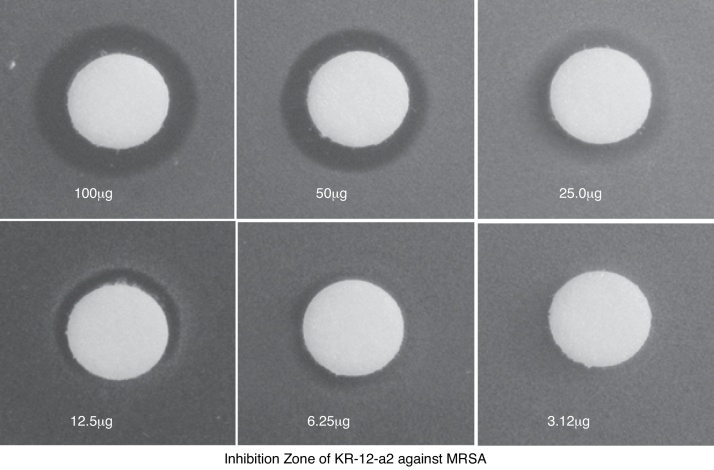
Inhibition zone test showing the antibacterial effect against MRSA. The size of the inhibition zone was dose-dependent.

KR-12-a2 produced an inhibition zone with the highest and lowest mean diameter against 100 μg and 6.25 μg MRSA, respectively (Fig. 2).

### ABR results

**Figure 3:**
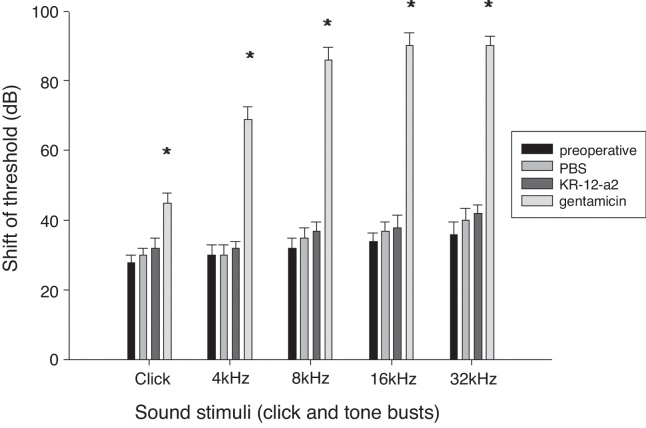
Hearing is preserved after topical application of KR-12-a2 as well as PBS, but shows deterioration after GM application. The GM group shows significant differences compared to the PBS or the KR-12-a2 group. Click (*p* = 0.014), 4 kHz (*p* = 0.011), 8 kHz (*p* = 0.009), 16 kHz (*p* = 0.003), 32 kHz (*p* = 0.002). There are no significant differences among preoperative, PBS and KR-12-a2 groups.

All the animals survived without complications or otitis media. There were no statistically significant differences in the ABR threshold before and after ventilation tube placement. The hearing threshold shift in the KR-12-a2 group and the PBS group was similar to that before the ventilation tube insertion. However, the gentamicin group showed an elevated hearing threshold and demonstrated statistically significant differences when compared to the PBS or the KR-12-a2 group (Fig. 3).

### SEM findings

**Figure 4:**
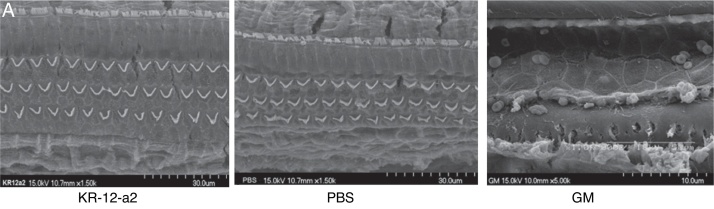

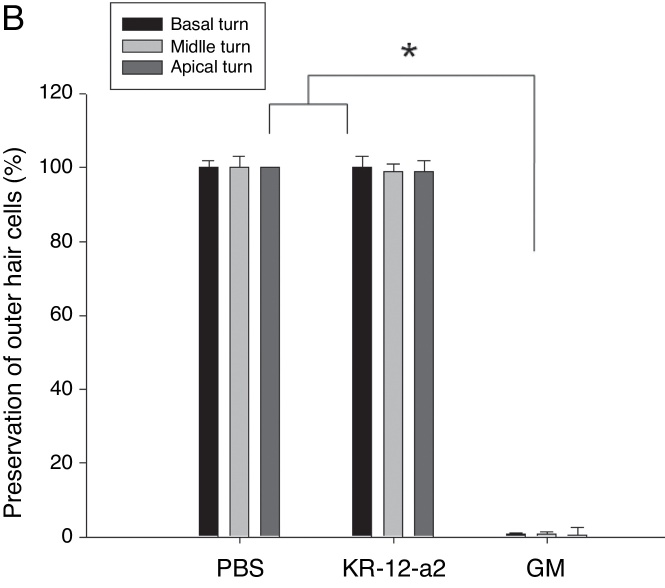
(A) Scanning electron microscopic findings showing well-preserved hair cells in the KR-12-a2 group; total hair cell loss is observed in the GM group. (B) There are no differences between the PBS and KR-12-a2 groups except with respect to the gentamicin results.

In the SEM findings, the KR-12-a2 group and PBS groups showed well-preserved Inner Hair Cells (IHC) and OHC in all turns. The findings showed intact IHC and OHC as in the PBS group. However, the gentamicin group showed total loss of OHC in basal turn, and nearly loss in the middle and apical turns (Fig. 4A–B).

## Discussion

The diagnosis of MRSA otorrhea should be considered in cases of persistent otorrhea in which standard ototopical and/or systemic antibiotic therapy fails. MRSA isolation rates among *S. aureus* ear infections have consistently increased up to 60.9%.^3^

Fishman et al. reported that in a large review of patients with culture-positive, persistent tympanostomy tube otorrhea, S. *aureus* accounted for 52% of the organisms cultured, of which 57% were MRSA.^20^ Presently, the most commonly used antibiotic against MRSA is vancomycin. Both laboratory experiments and clinical studies did not demonstrate significant ototoxicity after vancomycin treatment.^21^, ^22^, ^23^, ^24^ Although there is no emergence of VRSA in CSOM, new anti-MRSA peptides need to be studied for the prevention of VRSA or VISA. Mandal et al. reported that approximately 15% of MRSA strains were also resistant to vancomycin (VRSA).^25^ Recently, the topical application of daptomycin, a lipopeptide antibiotic that naturally occurs in the soil saprotroph *Streptomyces roseosporus* and that is useful in treating MRSA infections, revealed no ototoxicity in guinea pigs.^26^ However, the emergence of daptomycin-resistant MRSA has been already reported in other disease^27^ in addition to vancomycin resistance.^28^ Therefore, new AMP for MRSA otorrhea should be developed. The only peptide of the cathelicidin family that is found in the human body is LL-37, which has been shown to have broad antibacterial effects against both gram-positive and -negative bacteria.^29^ LL-37 is considered to play an important role in the first line of defense against local and systemic infection and in systemic invasion of pathogens at inflammation sites and wounds.^30^ Recently, Hou et al.^31^ reported that the antimicrobial peptide LL-37 and innate defense regulator peptide IDR-1 could ameliorate MRSA-induced pneumonia by exerting an anti-inflammatory property and attenuating pro-inflammatory cytokine release, thereby providing support for the hypothesis that both innate and synthetic peptides could play a role of protection against MRSA in vivo.

In the present study, we observed the antimicrobial activity of KR-12-a2 against MRSA. Inhibition zone and time-kill assays showed its potential antibacterial activity against MRSA. The present study investigated the ototoxicity by using topical application of KR-12-a2, GM, and PBS twice daily for 7 days. Hair cell damage was evaluated via ABR and SEM. There was no significant difference in the hearing threshold shift and hair cell preservation between the PBS and KR-12-a2 groups. However, the GM group showed total outer hair cell loss. Inner ear lesions occur predominantly in the basal turn, following the apical one with greater commitment of the first OHC row, with subsequent extension to IHC, stria vascularis, spiral ligament, and Reissner membrane.^32^ In this study, we evaluated only hair cells. In the GM group, the loss of OHC in basal turn than apical turn was observed. Gentamicin is highly water-soluble and has a polar nature that prevents it from readily crossing a membrane. A higher absorption rate would present the basal turn of the cochlea with a higher level of aminoglycoside prior to its equilibration into the perilymph.^33^ To date, ototoxicity induced by gentamicin depends on susceptibility, genetic predisposition, and caspase-dependent apoptotic cell death.^34^ The toxicity may be a dose-dependent phenomenon in the inner ear with higher levels resulting in marked hearing loss.^35^ In this study, we used a higher amount of GM than the dosage used by Kimura et al. Since the limitation of the present study is the absence of demonstrating therapeutic effects of KR-12-a2 in animal models, follow-up in vivo studies are considered necessary. Thus, future research will focus on the evaluation of the effects of topical KR-12-a2 by using a MRSA otitis animal model.

## Conclusion

In our experiment, topically applied KR-12-a2 solution did not cause hearing loss or cochlear damage in guinea pigs. The KR-12-a2 solution can be used as ototopical drops for treating MRSA otorrhea; however, further evaluations, such as the definition of optimal concentration and combination, are necessary.

## Conflicts of interest

The authors declare no conflicts of interest.
